# BASP1 interacts with oestrogen receptor *α* and modifies the tamoxifen response

**DOI:** 10.1038/cddis.2017.179

**Published:** 2017-05-11

**Authors:** Lindsey A Marsh, Samantha Carrera, Jayasha Shandilya, Kate J Heesom, Andrew D Davidson, Kathryn F Medler, Stefan GE Roberts

**Affiliations:** 1 School of Cellular and Molecular Medicine, University of Bristol, Bristol, UK; 2 Department of Biological Sciences, University at Buffalo, Buffalo, NY, USA; 3 Proteomics Facility, Faculty of Biomedical Sciences, University of Bristol, Bristol, UK

## Abstract

Tamoxifen binds to oestrogen receptor *α* (ER*α*) to elicit distinct responses that vary by cell/tissue type and status, but the factors that determine these differential effects are unknown. Here we report that the transcriptional corepressor BASP1 interacts with ER*α* and in breast cancer cells, this interaction is enhanced by tamoxifen. We find that BASP1 acts as a major selectivity factor in the transcriptional response of breast cancer cells to tamoxifen. In all, 40% of the genes that are regulated by tamoxifen in breast cancer cells are BASP1 dependent, including several genes that are associated with tamoxifen resistance. BASP1 elicits tumour-suppressor activity in breast cancer cells and enhances the antitumourigenic effects of tamoxifen treatment. Moreover, BASP1 is expressed in breast cancer tissue and is associated with increased patient survival. Our data have identified BASP1 as an ER*α* cofactor that has a central role in the transcriptional and antitumourigenic effects of tamoxifen.

BASP1 was originally identified as a membrane and cytoplasmic protein that sequesters lipids through an N-terminal myristoyl motif.^1^ BASP1 is also present in the nucleus and can function as a transcriptional corepressor for the Wilms’ tumour 1 protein WT1.^2^ BASP1 is recruited to the gene promoter by WT1 and converts WT1 from a transcriptional activator to a repressor. Intriguingly, the N-terminal myristoylation of BASP1 is required for its transcriptional corepressor activity.^3, 4^ Indeed, BASP1 acts as a corepressor through binding to the phospholipid PI4,5,P_2_ (PIP_2_) and recruiting histone deacetylase I (HDAC1) to the gene promoter. BASP1 can also block the transcriptional activation function of v-myc, but the mechanism by which it does this has not been explored.^5^

BASP1 is widely expressed in embryonic and adult tissues but its functions are as yet largely unknown.^1, 6^ The BASP1 gene is silenced in several tumour types including hepatocellular carcinoma, thyroid cancer and leukaemias.^7, 8, 9^ Moreover, cellular transformation by v-myc requires silencing of the BASP1 gene.^5^ Thus, current data suggest that BASP1 acts as a tumour suppressor. The inhibitory effect of BASP1 on WT1 transcription function is consistent with this notion because WT1 can act as an oncogene in many adult cancers.^10^ The widespread expression of BASP1 suggests that its transcriptional repressor activities are likely to be used by other DNA-binding transcriptional regulators that remain to be identified.

The oestrogen receptor *α* (ER*α*) belongs to a large family of nuclear hormone receptors that can act as either transcriptional activators or repressors in a ligand-dependent manner.^11^ ER*α* regulates a large number of genes that control proliferation, differentiation and other processes. Deregulated ER*α* is of particular concern in breast cancer and the majority of these tumours are exacerbated by ER*α*. Tamoxifen competes with oestrogen to interact with the ligand-binding domain of ER*α* and is widely used in breast cancer treatment.^12^ Although the mechanism of action of tamoxifen on ER*α* is complex, in breast cancer it generally drives the transcriptional repression activities of ER*α*. Many breast cancers can present tamoxifen resistance and several studies have identified the gene expression changes that accompany this resistance but the underlying reasons are still not clear.^12, 13, 14, 15, 16^

In this study, we identify components of the BASP1 complex and find several proteins that have previously been linked with ER*α*. We demonstrate that BASP1 interacts with ER*α*, the interaction is stimulated by tamoxifen and that BASP1 acts as a transcriptional coregulator for ER*α*. We find that BASP1 acts as a major selectivity factor in the transcriptional response to tamoxifen and that BASP1 enhances the antitumourigenic effects of tamoxifen. Moreover, BASP1 is expressed in breast cancer tissue and is associated with enhanced survival.

## Results

### ER*α* interacts with BASP1 and colocalises in the nucleus

Our previous studies showed that BASP1 is contained in large complexes within the nucleus.^4, 17^ We therefore sought to identify cofactors that bind to BASP1 using coimmunoprecipitation from nuclear extracts followed by mass spectrometry analysis. Chronic myelogenous leukaemia K562 cells do not express endogenous BASP1 and we utilised previously described K562 stable cell line derivatives that contain either pcDNA3 (V-K562) or pcDNA3 driving expression of BASP1 that contains a C-terminal HA tag (B-K562).^18^ Nuclear extracts were prepared from V-K562 and B-K562 cells and immunoprecipitation was performed with anti-HA antibodies. The immunoprecipitates were subjected to Orbitrap mass spectrometry analysis. The results revealed a high enrichment of components previously shown to be part of the nuclear actin network that binds ER*α*^19^ within the HA immunoprecipitates from B-K562 cells. These include *β*-actin, gelsolin, myosin-1-C (MYO1C) and flightless 1 (FLI1). We confirmed these findings by immunoprecipitation with HA antibodies using nuclear extracts prepared from V-K562 and B-K562 cells and immunoblotting directly for the identified factors (Supplementary Figure 1A).

**Figure 1:**
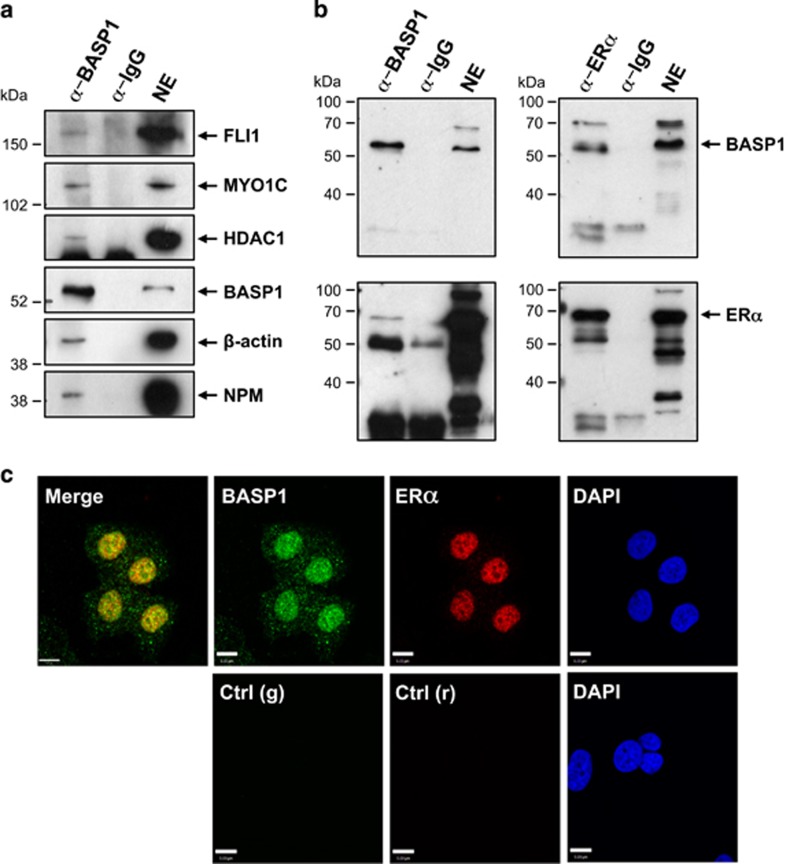
BASP1 associates with ER*α*. (**a**) Nuclear extracts (NE) from MCF7 cells were used in co-IPs using BASP1 or IgG antibodies. The samples were immunoblotted with antibodies against BASP1, HDAC1, *β*-actin, NPM, MYO1C and FLI1. (**b**) Co-IPs were performed from MCF7 nuclear extracts using BASP1 (left) or ER*α* (right) antibodies and immunoblotted with BASP1 (upper panels) and ER*α* (lower panels) antibodies. (**c**) MCF7 cells were used to perform coimmunoflourescence with rabbit BASP1 (green) and mouse ER*α* (red) antibodies. DAPI is shown. Control anti-rabbit (g) and anti-mouse (r) antibodies are shown below

The previous work that identified the nuclear actin network that binds ER*α* was performed with breast cancer MCF7 cells.^20^ MCF7 cells express BASP1 and it is largely localised to the nuclear fraction (Supplementary Figure 1B). We therefore sought to confirm the above finding in K562 cells with nuclear extracts prepared from MCF7 cells. Immunoprecipitation of endogenous BASP1 from MCF7 nuclear extracts, but not control IgG immunoprecipitates contained *β*-actin, nucleophosmin (NPM), MYO1C, FLI1 and, as previously shown, HDAC1 (Figure 1a).^3, 4^ These results raised the possibility that BASP1 can associate with ER*α*. We therefore used MCF7 nuclear extracts to perform immunoprecipitation with BASP1, ER*α* or control IgG antibodies. BASP1 immunoprecipitates contained ER*α*, and ER*α* immunoprecipitates contained BASP1 (Figure 1b). Moreover, immunofluorescence confirmed that BASP1 and ER*α* colocalise in the nucleus of MCF7 cells (Figure 1c). Taken together, the data in Figure 1 demonstrate that BASP1 and ER*α* associate with the same network of proteins within the nucleus of MCF7 cells.

### Tamoxifen enhances BASP1 interaction with ER*α*

**Figure 2:**
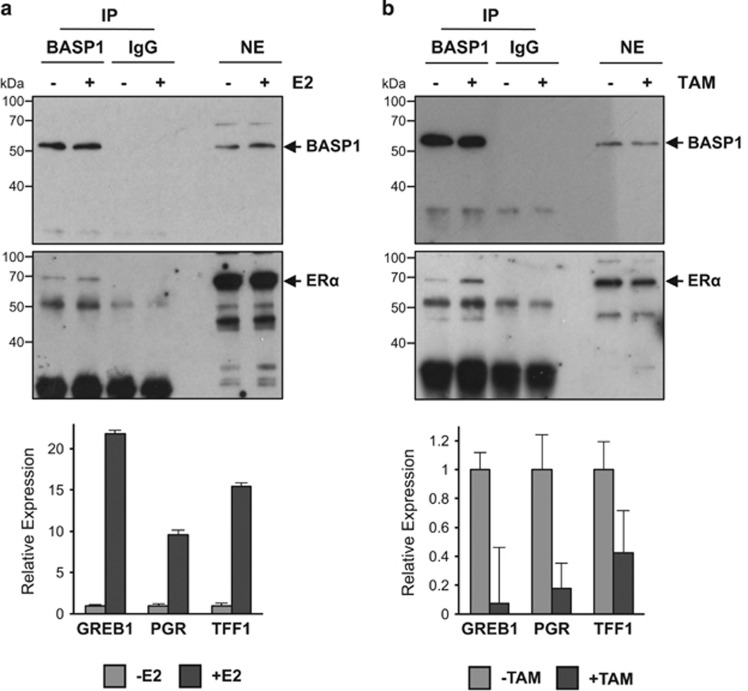
BASP1 association with ER*α* is enhanced by tamoxifen. (**a**) BASP1 and control IgG IP was performed using nuclear extracts from MCF7 cells that had been treated for 30 min with 10 nM estradiol (E2). Samples were probed with antibodies against BASP1 (upper panel) or ER*α* (lower panels). Immunoblots are representative of three independent experiments. RNA was extracted in parallel from MCF7 cells harvested after overnight treatment with E2 (**a**, lower panel). qPCR was performed to quantitate GREB1, PGR and TFF1 mRNA relative to GAPDH. Error bars are S.D. from the mean of three independent experiments. (**b**) As in part **a** except that 100 nM tamoxifen (TAM) was used to treat the cells

The function of ER*α* is regulated by its interaction with ligands. We therefore performed coimmunoprecipitation analysis with nuclear extracts derived from MCF7 cells after treatment with the ER ligand estradiol. MCF7 cells were hormone starved in phenol-red-free DMEM with 5% charcoal stripped foetal calf serum for 48 h before treatment with 10 nM estradiol (E2) or H_2_O control. Cells were harvested after 30 min of E2 treatment and used to prepare nuclear extracts. In tandem, MCF7 cells were treated identically but incubated for 24 h before preparation of RNA. The nuclear extracts were used for immunoprecipitation with anti-BASP1 antibodies and probed with ER*α* antibodies (Figure 2a). Equivalent amounts of ER*α* were immunoprecipitated in both control and E2-treated cells suggesting that the interaction between ER*α* and BASP1 is not regulated by estradiol. The RNA samples were used to prepare cDNA and analysed by qPCR to quantitate the expression of three ER*α* target genes (*GREB1*, *PGR* and *TFF1*). All three genes showed elevated expression in response to E2 confirming its activity (Figure 2a, below).

We next performed a similar experiment except that the MCF7 cells were seeded in normal DMEM and foetal calf serum before treatment with 100 nM tamoxifen or (control) ethanol for the same time points as above (30 min for nuclear extract preparation and 24 h for RNA preparation). Anti-BASP1 immunoprecipitates from cells treated with tamoxifen showed that more ER*α* was contained within anti-BASP1 immunoprecipitates from nuclear lysates after tamoxifen treatment compared with untreated cells (Figure 2b). Analysis of the RNA showed that tamoxifen treatment induced transcriptional repression of ER*α* target genes confirming activity of the drug (Figure 2b, below). Analysis of another ER-positive breast cancer cell line (T47D) confirmed that BASP1 immunoprecipitates contained more ER*α* after treatment of the cells with tamoxifen (Supplementary Figure 2). Taken together, the data in Figure 2 and Supplementary Figure 2 suggest that BASP1 associates preferentially with the tamoxifen-liganded ER*α* and is consistent with the possibility that BASP1 is a transcriptional corepressor of ER*α*.

### ER*α* and BASP1 regulate a subset of ER*α* target genes

**Figure 3:**
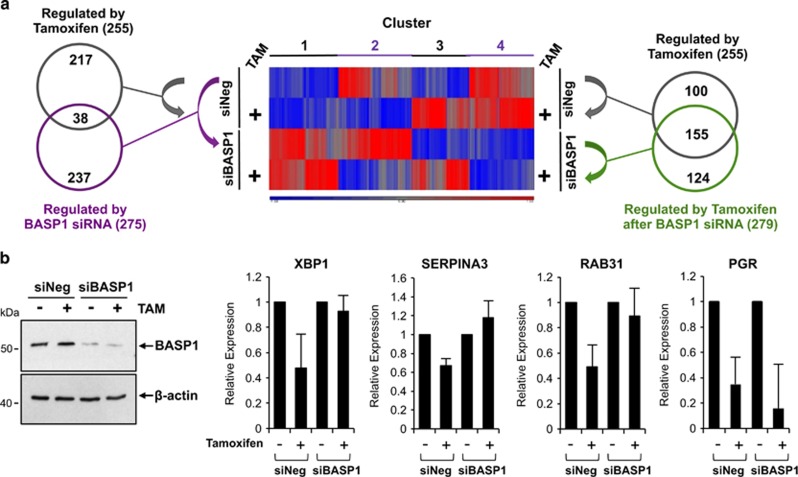
BASP1 regulates ER*α* target genes and modifies the tamoxifen response. (**a**) Total RNA-sequencing analysis was performed on RNA extracted from MCF7 cells transfected with either control siRNA (siNEG) or BASP1 siRNA (siBASP1) after overnight treatment with 100 nM tamoxifen (+TAM) or vehicle control (-TAM). The heatmap at centre shows tamoxifen-responsive genes (±TAM) in knockdown BASP1 (siBASP1) and negative control (siNEG) MCF7 cells. The Venn diagram at left shows the number of targets significantly and 1.5-fold altered by tamoxifen (grey circle) and by BASP1 siRNA (purple circle). The Venn diagram at right compares the tamoxifen-responsive target genes in the presence (siNEG; grey circle) and absence (siBASP1; green circle) of BASP1. (**b**) RNA prepared from MCF7 cells treated as above and used for qPCR to detect XBP1, SERPINA3, RAB31 and PGR mRNA relative to GAPDH mRNA. Error bars are S.D. from the mean of three independent experiments. At left, MCF7 whole-cell extracts were immunoblotted in parallel with anti-BASP1 and anti-*β*-actin antibodies

Our data so far suggest that BASP1 can act as a transcriptional coregulator for ER*α* and that their cooperation may be enhanced by the treatment of cells with tamoxifen. To further explore a functional role that BASP1 has in ER*α*-mediated transcriptional regulation, we next sought to identify genes that are regulated by BASP1 and ER*α*. MCF7 cells were transfected with control (siNEG) or BASP1 siRNA (siBASP1) and 24 h later treated with 100 nM tamoxifen or control drug vehicle. Forty-eight hours after initial transfection, RNA was prepared and subjected to total RNA sequencing. Figure 3a (centre) shows a heatmap of the transcripts with a statistically significant change (>1.5-fold) between at least two pairwise comparisons (full data heatmap gene list is in Supplementary Table 1). Genes within the heatmap can be divided into four distinct clusters: cluster 1 contains genes that are generally repressed by BASP1 but not altered by tamoxifen, cluster 2 contains genes that are repressed by tamoxifen either with or without BASP1, cluster 3 are genes activated by tamoxifen and cluster 4 are genes that are generally activated by BASP1. At left, a Venn diagram compares the 255 genes regulated by tamoxifen in MCF7 cells transfected with control siRNA (grey circle) with the 275 transcript changes that occurred when MCF7 cells were transfected with BASP1 siRNA in the absence of tamoxifen (purple circle). Significantly, these two groups contained 38 genes in common. Thus, 15% of the genes that are regulated by tamoxifen are also independently regulated by BASP1. These data demonstrate that BASP1 regulates the expression of genes that are also regulated by ER*α*. Indeed, this group contains several known ER*α* target genes including *BMPER, FHL1* and *INHBB*^15, 21, 22^ (see Supplementary Table 2).

The interaction between ER*α* and BASP1 is enhanced when MCF7 cells are treated with tamoxifen. We were therefore interested to compare the effect of tamoxifen on the transcriptome in control siRNA cells (the 255 genes as above) with the genes that are regulated by tamoxifen when the cells were depleted of BASP1 by siRNA (green circle, 279 genes; Figure 3a, at right). This comparison revealed that the effect of tamoxifen on the transcriptome is significantly altered in MCF7 cells when BASP1 is depleted. Indeed, only 155 (of 255) genes that are regulated by tamoxifen in control siRNA MCF7 cells are still regulated by tamoxifen in cells depleted of BASP1. These genes include classic ER*α* target genes such as *PGR, GREB1* and *TFF1*. However, 100 of the genes that are regulated by tamoxifen in cells transfected with control siRNA are no longer regulated by tamoxifen when BASP1 is depleted (see Supplementary Table 3). Thus, 40% of the genes that are regulated by tamoxifen in MCF7 cells are dependent on BASP1. Several of these genes have reported roles in tamoxifen resistance including *DLC1, BMPER* and *SOX2*.^14, 15^ Interestingly, we found that 124 genes were regulated by tamoxifen in cells depleted of BASP1 that were not regulated by tamoxifen in cells that had been transfected with control siRNA (Supplementary Table 4). Of these 124 genes, 64 become repressed and 60 activated by tamoxifen in the absence of BASP1. DAVID analysis^23^ of these novel tamoxifen targets revealed that the activated genes are involved in cell cycle, signalling and negative regulation of apoptosis, such as *BCL3*.^24, 25^ Novel tamoxifen-repressed targets generally have a role in chromatin and nucleic acid regulation. In summary, BASP1 is required for the regulation of over one-third (100/255) of the genes targeted by tamoxifen in MCF7 cells. Furthermore, BASP1 inhibits the effect of tamoxifen in the regulation of 124 genes. We conclude that BASP1 has a major role in the transcriptional response of MCF7 cells to tamoxifen.

Our results so far have showed that BASP1 is an ER*α*-interacting protein and that their association is stimulated by the treatment of cells with tamoxifen. In addition, BASP1 regulates several ER*α* target genes and also modifies the tamoxifen response of MCF7 cells. We confirmed the BASP1-dependent modification of the tamoxifen response of the ER*α* target genes *XBP1*,^19^ *SERPINA3*^15, 26^ and *RAB31*^27^ by direct qPCR (Figure 3b). In addition, we analysed expression of the *PGR* gene, which is repressed by tamoxifen and was not regulated by BASP1 in the RNAseq data. We also confirmed the BASP1-dependent repression of *XBP1* and *RAB31* expression by transfection of BASP1 into T47D cells, which enhanced the repressive effect of tamoxifen (Supplementary Figure 3). Taken together, the data in Figure 3 and Supplementary Figure 3 suggest that BASP1 acts as a gene-specific transcriptional coregulator of ER*α* and that BASP1 is required for the tamoxifen-dependent transcriptional repression of a significant number of ER*α* target genes.

### BASP1 is recruited to the promoter region of ER*α* target genes to regulate transcription

**Figure 4:**
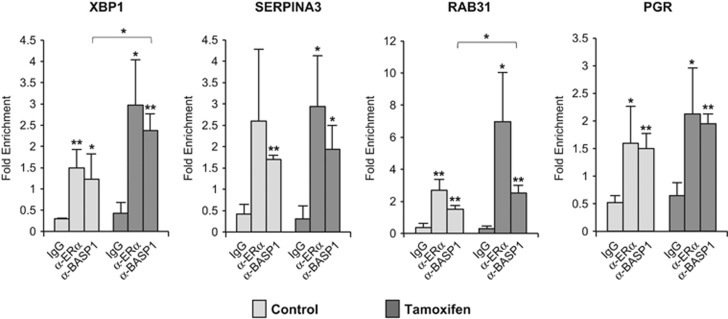
BASP1 localises to the promoter region of genes regulated by ER*α*. MCF7 cells were subjected to ChIP assay with control IgG, BASP1 or ER*α* antibodies. Enrichment at the ER*α*-binding sites in the XBP1, SERPINA3, RAB31 and PGR promoters are presented relative to a control gene region. Error bars are S.D. from the mean of three independent experiments; *P<0.05 and **P<0.01 is Student's *t*-test

BASP1 regulates the transcriptional activity of WT1 by recruitment to the WT1-binding site of its target genes.^2^ We therefore next used chromatin immunoprecipitation (ChIP) to determine if BASP1 and ER*α* occupy the promoters of the ER*α* target genes that we analysed in Figure 3b. ChIP was performed with control IgG, ER*α* and BASP1 antibodies using fragmented, cross-linked chromatin from MCF7 cells that had either been treated with either tamoxifen or mock treated for 16 h. Figure 4 shows that BASP1 and ER*α* are recruited to the *XBP1, SERPINA3* and *RAB31* promoters. At the *XBP1* and *RAB31* promoters, we observed a significant increase in BASP1 ChIP in the cells that had been treated with tamoxifen, which is consistent with the tamoxifen-enhanced coimmunoprecipitation of ER*α* and BASP1 (Figure 2b). We also note that BASP1 was detected at the *PGR* promoter although the *PGR* gene is not altered when BASP1 expression is reduced by siRNA (Figure 4).

### BASP1 acts as a tumour suppressor in breast cancer MCF7 cells

**Figure 5:**
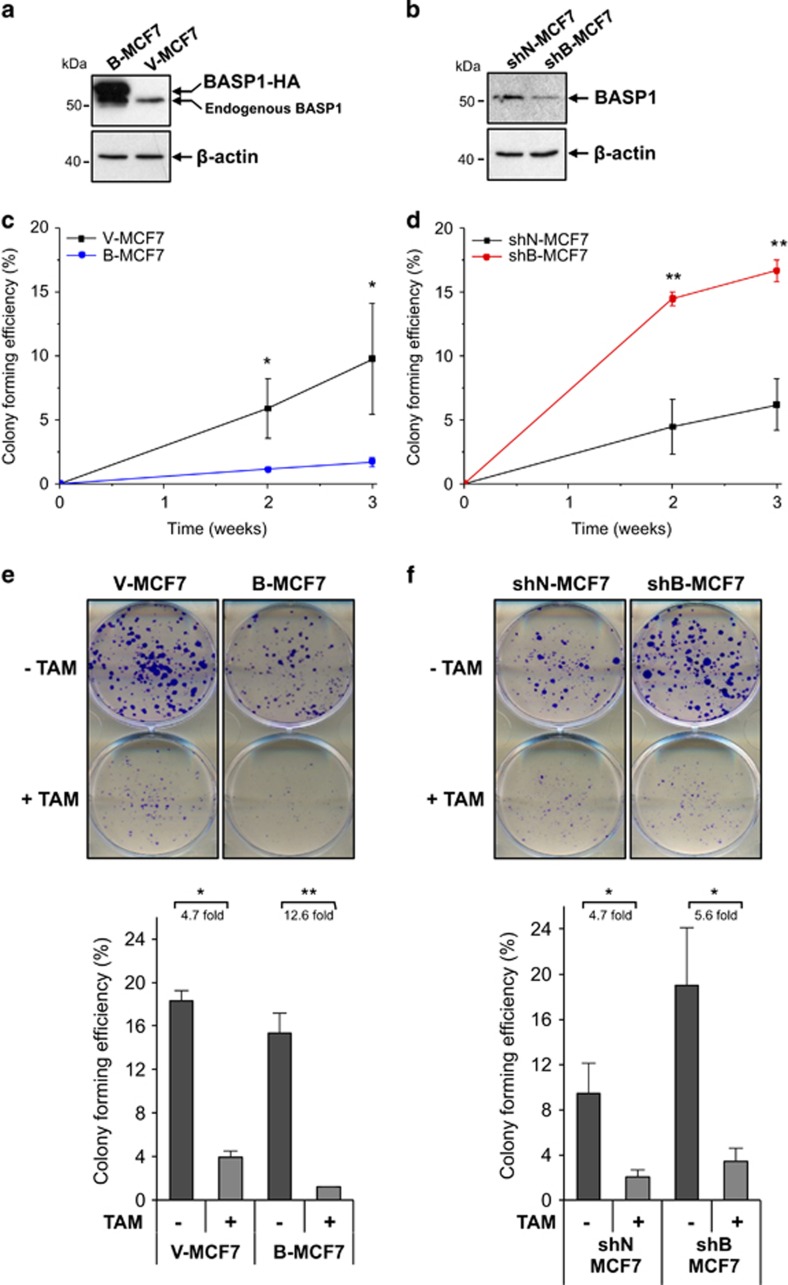
BASP1 has a tumour-suppressor role in MCF7 cells. (**a**) Whole-cell extracts prepared from V-MCF7 and B-MCF7 cells were immunoblotted with BASP1 or *β*-actin antibodies. (**b**) The stable clones were subjected to soft agar colony formation assays. Error bars are S.D. from the mean of three independent experiments. A repeated-measures two-way ANOVA with a Bonferroni’s *post-hoc* analysis and a Student's *t*-test was performed for each time point. The*t*-test values are shown (*P<0.05, **P<0.01). The ANOVA showed that for both the 2- and 3-week time points there was a significant difference between V-MCF7 and B-MCF7 cells. (**c**) As in part **a** except that stable MCF7 cells were control pSilencer (shN-MCF7) and pSilencer-BASP1 (shB-MCF7) (**d**). As in part **b** except that shN-MCF7 and shB-MCF7 were compared. (**e**) V-MCF7 and B-MCF7 cells were seeded into six-well plates at 500 cells per well and subjected to a 72-h treatment of 100 nM tamoxifen (+TAM) or vehicle control (-TAM) 48 h after seeding. Cells were stained with crystal violet after 9 more days. Mean colony formation efficiency (%) was calculated from triplicate wells for each cell line. Error bars are S.D. from the mean of three independent experiments; *P<0.05 and **P<0.01 obtained by Student's *t*-test. (**f**) As in part **e** except that shN-MCF7 and sh-MCF7 cells were used

Our results so far suggest that BASP1 is a transcriptional coregulator of ER*α* and that it preferentially acts on ER*α* in the presence of tamoxifen to regulate a subset of ER*α* target genes. Tamoxifen acts as an anti-oestrogen in breast cancer and inhibits growth. We therefore considered that BASP1 may regulate the tumourigenicity of MCF7 cells. We generated a stable MCF7 cell line derivative that overexpresses C-terminally HA-tagged BASP1 by transfecting MCF7 cells with empty pcDNA3 (V-MCF7) and pcDNA3-BASP1 (B-MCF7). Our previous studies have used this same tagged BASP1 derivative to study its corepressor activity on WT1.^3, 4, 17^ Immunoblotting of the stable cell lines showed that B-MCF7 cells, but not the control V-MCF7 cells, ectopically express the tagged BASP1 (Figure 5a). We subjected the MCF7 stable clone cells to soft agar colony formation assays, grown for 3 weeks and counted at weekly intervals to calculate average colony formation efficiency (%CFE). The results revealed that B-MCF7 cells had a significantly lower %CFE compared with control V-MCF7s (Figure 5b). We next generated stable MCF7 cell line derivatives that contain the pSilencer vector driving control shRNA (shN-MCF7) or a BASP1-specific shRNA that we have used before (shB-MCF7).^6^ Immunoblotting of whole-cell extracts confirmed that the shB-MCF7 cells contained reduced levels of BASP1 (Figure 5c). Soft agar colony formation assays over a 3-week period revealed that the shB-MCF7 cells had a significantly higher %CFE when compared with the control shN-MCF7 cells (Figure 5d). Comparable results were obtained from soft agar assays seeded with MCF7 cells 24 h after transient transfection with control and a different BASP1 siRNA (Supplementary Figure 4). Taken together, the data in Figures 5a and d and Supplementary Figure 4 demonstrate that BASP1 suppresses the tumourigenicity of MCF7 cells.

The results from Figure 2b and Figure 4 revealed that the ER*α*–BASP1 interaction is augmented in cells that have been treated with tamoxifen. We therefore next investigated the effect of tamoxifen on colony formation in the MCF7 cell line derivatives used above. The B-MCF7 cells and V-MCF7 cells were seeded in triplicate to six-well plates at 500 cells per well, then treated with and without 100 nM tamoxifen over 48 h. After 72 h, fresh media were added and cells were grown for 9 more days before fixation and crystal violet staining. Cells were counted and the average %CFE was calculated. Consistent with the results from the soft agar assays, B-MCF7 cells grew smaller and less colonies compared with V-MCF7 control cells (Figure 5e, quantitation is shown below the photograph). Significantly, although tamoxifen treatment of V-MCF7 cells elicited a 4.7-fold reduction in %CFE, there was a 12.6-fold reduction in %CFE in the B-MCF7 cells treated with tamoxifen. Moreover, overexpression of BASP1 in T47D cells led to a similar effect in enhancing the effect of tamoxifen in reducing colony formation (Supplementary Figure 5). Analysis of the shN-MCF7 and shB-MCF7 cells showed that the shB-MCF7 cells grew larger and more colonies compared with shN-MCF7 control cells (Figure 5f). Overall, the effect of tamoxifen on %CFE was comparable with shN-MCF7 and shB-MCF7 cells. Taken together, the data in Figure 5 and Supplementary Figures 4 and 5 demonstrate that BASP1 suppresses the tumourigenicity of breast cancer cells. Furthermore, overexpression of BASP1 in both MCF7 and T47D cells enhances the anticancer activity of tamoxifen.

### A role for BASP1 in breast cancer

**Figure 6:**
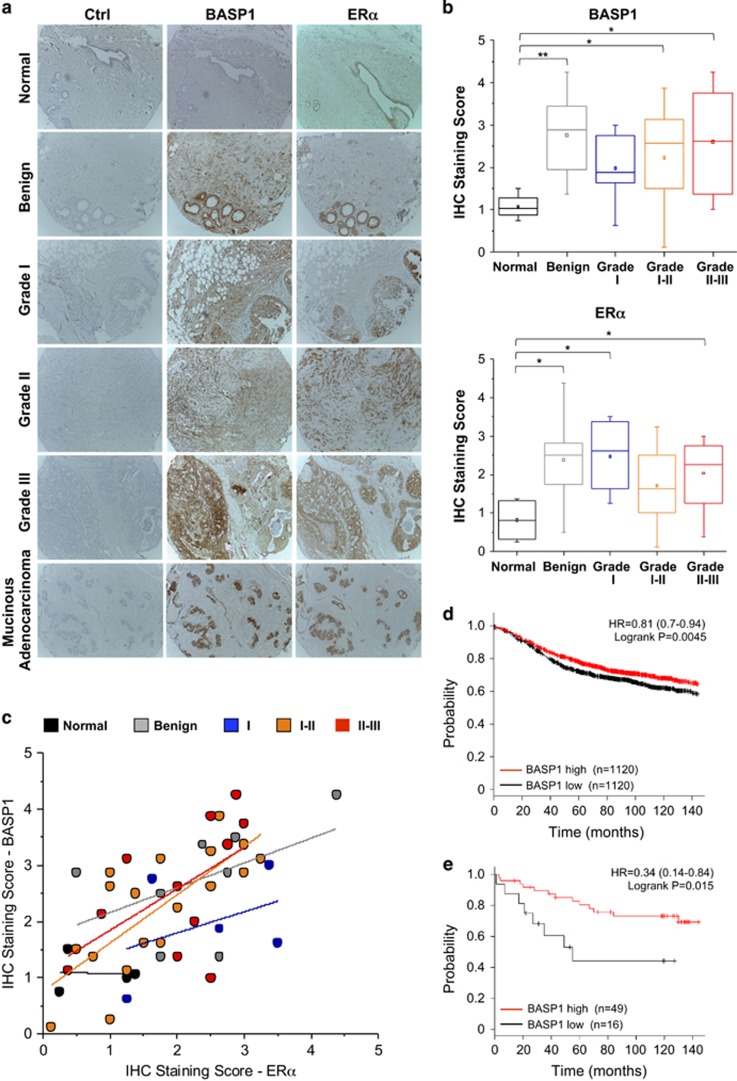
BASP1 is expressed in human breast cancer. (**a**) Representative images of IHC stained breast cancer tissue samples of each cancer subtype stained using BASP1 and ER*α* antibodies, and control samples (Ctrl). Benign tumour sample has fibrotic changes. Grade I tumour is a ductal carcinoma *in situ* and grade II and III are invasive ductal cell carcinomas. Samples were scored for BASP1 and ER*α* between 0 (no expression) and 5 (high expression) independently by four observers. (**b**) Box and whisker plots of median BASP1 (upper panel) and ER*α* scores by subtype. Data were analysed by one-way ANOVA with Bonferroni’s *post-hoc* analysis to show that the level of BASP1 in the normal samples was significantly less than the benign sample or tumours. *T*-test values are shown (*P<0.05, **P<0.01). (**c**) Scatter plot of mean BASP1 and ER*α* IHC scores for individual normal and tumour samples with a best line fit for each category. (**d**) Kalpan–Meier plot of ER-positive breast cancer analysing BASP1 expression against probability of relapse-free survival (*n*=2240). (**e**) Kaplan–Meier plot of ER-positive breast cancer patients that were treated with tamoxifen alone comparing BASP1 expression against probability of overall survival (*n*=65)

Our results so far suggest that BASP1 can function as a transcriptional corepressor for ER*α* and acts as a tumour suppressor in MCF7 cells. We next analysed a breast cancer tissue array to determine BASP1 and ER*α* expression by immunohistochemistry (IHC). Examples of normal, benign and cancer tissues are shown in Figure 6a and average quantitation across all samples is shown in Figure 6b. BASP1 and ER*α* showed a low level of staining in normal tissue, but both were significantly elevated in benign and malignant tissue. Indeed, there was a general correlation between the expression of ER*α* and BASP1 across the samples, whereas the benign tumours were most likely to express the highest level of BASP1 (Figure 6b). This was confirmed in a scatter plot of ER*α* and BASP1 IHC staining score for each of the samples, colour coded and with a best-fit line for each stage (Figure 6c). Interestingly, stage I–II (orange) and II–III (red) tumours show a steeper slope than the benign (grey) and grade I (blue) tumours suggesting that the more severe tumours exhibit a lag in BASP1 expression compared with ER*α*. Analysis of BASP1 expression in ER-positive breast cancer by Kaplan–Meier Plotter^28^ revealed a modest but significant correlation between high BASP1 expression and survival (Figure 6d). We next interrogated this data set to exclusively analyse patients that had only been treated with tamoxifen. Although the patient number is small (65), there is a significant and robust increase in survival in those patients that express high BASP1 (Figure 6e). Taken together, these results suggest that expression of BASP1 in breast cancer tissue is related to ER*α* expression and that BASP1 likely acts as a tumour suppressor in breast cancer.

## Discussion

In this study, we have demonstrated that BASP1 can act as a transcriptional corepressor of ER*α*. BASP1 is recruited to the promoter regions of ER*α* target genes and its functional interaction is enhanced by the treatment of cells with tamoxifen. BASP1 appears to be selective in the ER*α* target genes that it regulates. For example, the *GREB1*, *PGR* and *TFF1* genes, which are frequently studied as ER*α* target genes, do not appear to be regulated by BASP1 in either the absence or presence of tamoxifen. Even so, we detected BASP1 at the ER*α*-binding region of the *PGR* gene, suggesting that recruitment of BASP1 along with ER*α* is not the only determinant of their functional interplay and that other conditions are necessary.

Our RNAseq analysis revealed that depletion of BASP1 in MCF7 cells caused a major change to the transcriptional response to tamoxifen. Several genes associated with tamoxifen resistance including *SERPINA3, RAB31, XBP1, BMPER, SOX2* and *DLC1* were dependent on BASP1 for their regulation by tamoxifen. Lack of repression of these gene targets in response to tamoxifen leads to sustained tumour growth in breast cancer.^14, 15^ In addition, we note that the expression of 124 genes became tamoxifen-dependent only when BASP1 expression was reduced by siRNA. Overall, ablation of BASP1 expression modified the expression of 40% of the genes that were normally regulated by tamoxifen in MCF7 cells.

Previous studies have reported that BASP1 can act as a tumour suppressor and our data here found that BASP1 can inhibit the tumourigenicity of MCF7 cells. Moreover, consistent with the BASP1-dependent modification of the transcriptional response to tamoxifen, overexpression of BASP1 in MCF7 cells significantly enhanced the effect of tamoxifen on colony formation. Our histological analysis found that BASP1 is not expressed in normal breast tissue but is frequently present at all stages of breast cancer, but particularly in the benign tumours. Previous studies reported that BASP1 is highly expressed in the terminal end buds of the developing mammary ducts during puberty.^29^ It is likely that the expression of BASP1 is reactivated in adult breast epithelia as an early response to cellular transformation and that it then suppresses cell growth. This is consistent with the enhanced survival of breast cancer patients with higher levels of tumour BASP1. Further studies will be required to determine if BASP1 can be used as a marker for patients that respond optimally to tamoxifen treatment. It will also be interesting to determine if BASP1 affects the response of ER*α* to other therapeutic agents that target this transcription factor.

The transcriptional corepressor function of BASP1 was first identified in the context of WT1.^6^ BASP1 can also inhibit the transcriptional activation function of v-myc, but the mechanism is not yet known.^5^ Our finding that BASP1 can act as a transcriptional corepressor for ER*α* suggests that BASP1 might act as a cofactor for several transcriptional regulators. As a cofactor for WT1, BASP1 recruits HDAC1 and the ATP-dependent chromatin remodeler BRG1.^3, 4^ These histone modifiers are also known to function with ER*α*.^30, 31, 32^ BASP1 requires an N-terminal myristoyl motif that interacts with nuclear PIP_2_ to facilitate interactions with HDAC1 and BRG1.^3, 4^ Lipidation of BASP1 is also required for its association with membranes and BASP1 is able to promote the formation of lipid rafts.^33, 34^ It is therefore of interest that ER*α* associates with lipid rafts where it is suggested to regulate cellular signalling.^35^ It will be interesting to determine if BASP1 also has a role in this ER*α* function and if this is connected with the nuclear events that we report here.

## Materials and methods

### Cells, stable clones, antibodies and siRNAs

MCF7 and T47D cells were purchased from the European Collection of Authenticated Cell Cultures (Salisbury, UK). K562 cells were cultured in RPMI medium and MCF7 cells in Dulbecco’s modified Eagle’s medium. Stable K562 cell lines were described before.^17^ Stable MCF7 cell lines were transfected with either pcDNA3 or pSilencer vectors as previously described^6^ using Lipofectamine (Thermo Fisher, Paisley, UK), 48 h after seeding. Media were changed to DMEM containing 140 *μ*g/ml G418 72 h after transfection and single colonies cultured 3 weeks later. BASP1 siRNAs were as described in Toska *et al.*^4^ ER*α* (sc-8005) and ER*α* HC-20 (sc-543) antibodies were from Santa Cruz. Gelsolin (Santa Cruz, CA, USA, G4896), *β*-actin (A5316) and MYO1C (M3567) antibodies were from Sigma Aldrich (St Louis, MO, USA). FlI-1 (ab28089) and NPM (ab52644) antibodies were from Abcam (Cambridge, UK). HDAC1 (10E2) and HA antibodies were purchased from Cell Signalling (Leiden, Holland).

### Protein, RNA and ChIP analysis

Immunoprecipitation was performed using BASP1 antibodies and Dynabeads (Thermo Fisher) as previously described.^6^ Mass spectrometry analysis of immunoprecipitates was as described.^36^ RNA was extracted and analysed as before^3^ using the following primers: PGR: fwd, 5′-CGCGCTCTACCCTGCACT-3′, rev, 5′-TGAATCCGGCCTCAGGTAGTT-3′; GREB1: fwd, 5′-CAAAGAATAACCTGTTGGCCCTGC-3′, rev, 5′-GACATGCCTGCGCTCTCATACTTA-3′; TFF1: fwd, 5′-CATCGACGTCCCTCCAGAAGAG-3′, rev, 5′-CTCTGGGACTAATCACCGTGCTG-3′ RAB31: fwd, 5′-CGAGCACATGATGGCGATACG-3′, rev, 5′-GTCCTTCAGCAGTGCACAGGA-3′ SERPINA3: fwd, 5′-CTGACCTGTCAGGGATCACA-3′, rev, 5′-TGCAGAAAGGAGGGTGATTT-3′ XBP1: fwd, 5′-GCGCCTCACGCACCTG-3′, rev, 5′-GCTGCTACTCTGTTTTTCAGTTTCC-3′ GAPDH: fwd, 5′-GAAATCCCATCACCATCTTCCAGG-3′, rev, 5′-GAGCCCCAGCCTTCTCCATG-3′. RNA samples were subjected to library preparation using standard Illumina protocols (TruSeq, Illumina, San Diego, CA, USA). RNAs were pair-end sequenced using a HiSeq 2500 (Illumina) and HighSeq Reagents 4 software. Data were imported into online Galaxy NGS software suite^37^ and mapped using TopHat^38^ to the human genome (hg19). We used uniquely mapped reads for gene expression analysis with Cufflinks using the Ensembl human gene annotation as a guide (v.64), and Cuffdiff to obtain differentially expressed transcripts.^39^ ChIP assays were performed as before^3^ using the following primers: PGR: fwd, 5′-AATGAGGCTGACATTCTGGGA-3′, rev, 5′-GTTGACCTCATTCCAAGGCAG-3′ RAB31: fwd, 5′-CACCTGACACCAATCCTTTGTG-3′, rev, 5′-CCAGAACAAGTAGACAGCTCTC-3′ SERPINA3: fwd, 5′-CAACAAGAGGTGACTGTGTGG-3′, rev, 5′-GACCTGCAGATTGAGTGCAGA-3′ XBP1: fwd, 5′-CTGGAACAAAATTCCCAGCA-3′, rev, 5′-ATTCAAACCCTGCCCCTAGA-3′ Control: fwd, 5′-CTGACGGCAACTTCAAC-3′, rev, 5′-GGTGCACAGGGCCTT-3′.

### Colony formation and soft agar assays

Soft agar assays on MCF7 cells were performed as described.^40^ For plate colony formation assays, MCF7 cells were seeded in triplicate to six-well plates at 500 cells per well. After 48 h, cells were treated for 72 h with 100 nM tamoxifen (Sigma, St Louis, MO, USA) or vehicle control. Cells were grown for a further 9 days before fixation in 4% formaldehyde and stained for 10 min using crystal violet.

### IHC and immunofluorescence

Breast cancer tissue arrays (Abcam, ab178111) were subjected to IHC with BASP1 antibody (1:100) or anti-ER*α* antibody (1:500) as described.^41^ Sections were graded between 0 (background) and 5 (strongly positive) by four independent observers). For immunofluorescence, MCF7 cells grown on coverslips were incubated with CSK buffer (10 mM PIPES pH 7.0, 100 mM NaCl, 300 mM sucrose, 3 mM MgCl_2_, 0.5% Triton X-100) for 10 min. Cells were fixed with 4% paraformaldehyde, permeabilised with 0.1% Triton X-100 and washed with phosphate-buffered saline (PBS). Cells were blocked for 1 h in 1% BSA and then incubated with primary antibodies for 3 h (BASP1 rabbit 1:50, ER*α* mouse sc-8005 1:200). Cells were washed with PBS and then incubated for 45 with Dylight 488 anti-rabbit (1 : 250) and DyLight 594 goat anti-mouse (1:250) antibodies. Nuclei were counterstained with DAPI (1*μ*g/ml).

### Major data sets

The RNAseq data presented in Figure 2a is available at the NCBI Gene Expression Omnibus (accession no. GSE78199).
