# Supplementary material for: BASP1 interacts with oestrogen receptor α and modifies the tamoxifen response
Source: Cell Death Dis. 2017 May 11;8(5):e2771–. doi: 10.1038/cddis.2017.179 (PMC5520704; doi:10.1038/cddis.2017.179)
Supplement: Supplementary Figures [file cddis2017179x1.pdf]

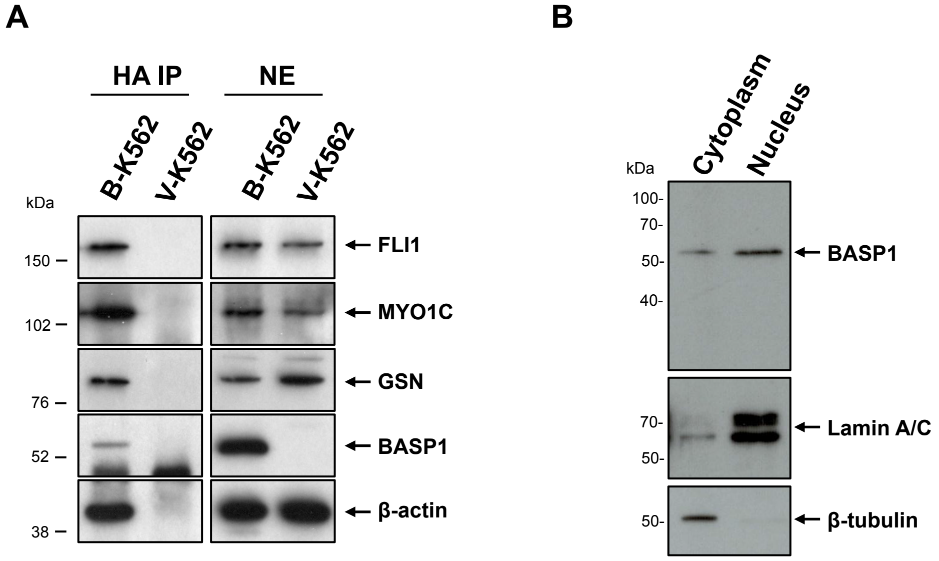

Supplementary Figure. 1. BASP1 interacts with several components of the ERα nuclear actin network and is nuclear in MCF7 cells.

(A) Immunoprecipitation was performed with anti-HA antibodies using nuclear extracts prepared from control K562 cells (V-K562) and K562 cells that express a C-terminally HA-tagged BASP1 derivative (B-K562). The immunoprecipitates were the immunoblotted with antibodies against Flightless 1 (FLI1), Myosin-1-C (MYO1C), gelsolin (GSN), BASP1 and β-actin. Molecular weigh markers (kDa) are shown at left. NE is nuclear extract. (B) Nuclear and cytoplasmic extracts were prepared from MCF7 cells and then immunoblotted to detect BASP1, Lamin A/C and β-tubulin. Molecular weigh markers (kDa) are shown at left.

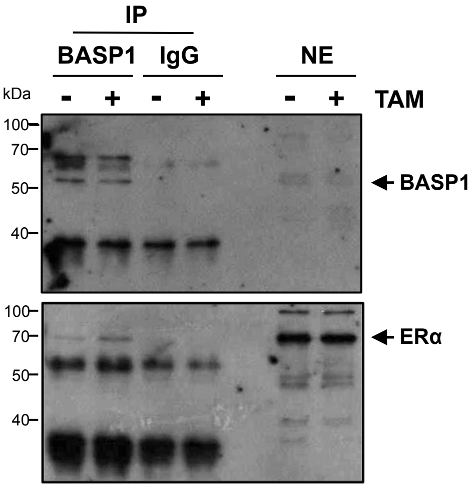

Supplementary Figure 2. BASP1 association with ERα is enhanced by Tamoxifen treatment of T47D cells.
BASP1 and control IgG IP was performed using nuclear extracts (NE) from T47D cells that had been treated for 30 minutes with 100nM tamoxifen (TAM, +) or vehicle control (-) . Samples were probed with antibodies against BASP1 (upper panel) or ERα (lower panels). Molecular weight markers are shown at left.

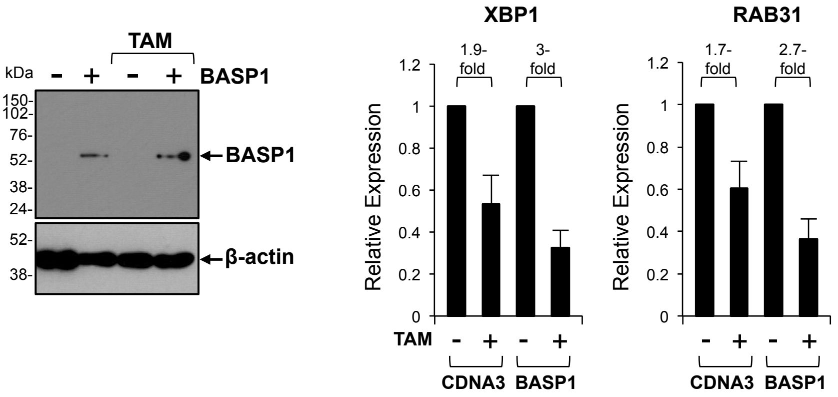

Supplementary Figure 3. BASP1 regulates ERα target genes and modifies the tamoxifen response in T47D cells.
T47D cells transfected with either control CDNA3 vector (-) or the same vector driving expression of BASP1 were subjected to overnight treatment with 100nM tamoxifen (TAM, +) or vehicle control (-). At left, whole cell extracts were immunoblotted with anti-BASP1 and anti-β-actin antibodies. RNA was prepared from T47D cells treated as above and used for q-PCR to detect XBP1 and RAB31 mRNA relative to GAPDH mRNA. Error bars are SDM of three independent experiments. The fold-inhibition of each gene by Tamoxifen in control- or BASP1-transfected cells is shown above the bars.

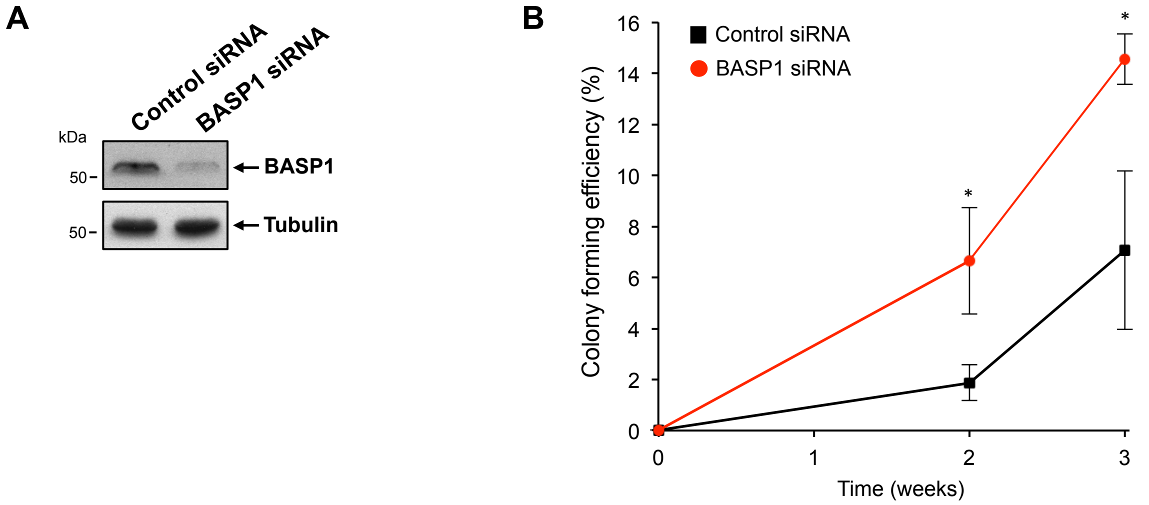

Supplementary Figure 4. Transient knockdown of BASP1 increases the colony forming efficiency of MCF7 cells

(A) MCF7 cells were transfected with either control siRNA or BASP1 siRNA. 48 hours after transfection, whole cell extracts were immunoblotted with anti-BASP1 (upper panel) and anti-Tubulin (lower panel) antibodies. Molecular weigh markers (kDa) are shown at left. (B) Cells were transfected as in part A and subjected to soft agar colony formation assays 24 hours after transfection. Error bars are obtained from standard deviation from the mean (SDM) of three independent experiments. A repeated measures two-way ANOVA with a Bonferroni's post-hoc analysis and a Student's t test was performed for each time point. The t-test values are shown (* = p<0.05). The ANOVA showed that for both the 2 and 3 week time points there was a significant difference between BASP1 siRNA and control siRNA.

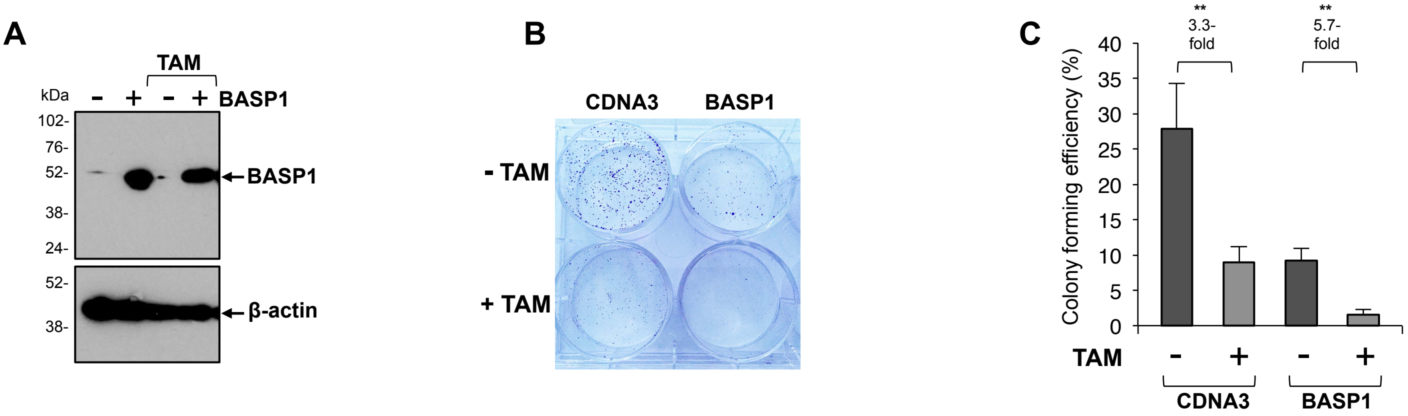

Supplementary Figure 5: Overexpression of BASP1 in T47D cells decreases colony forming efficiency and enhances the effect of Tamoxifen

T47D cells were transfected with either empty CDNA3 vector (-) or the same vector driving the expression of BASP1. (A) 48 hours later a sampe of cells was taken for preparation of whole cell extracts followed by immunoblotting with either BASP1 antibodies or β-actin antibodies. (B) The remaining cells, seeded into 6 well plates at 1000 cells per well, were subjected to a 72 hour treatment with 100nM tamoxifen (+TAM) or vehicle control (-TAM). Cells were stained with crystal violet after 9 more days. (C) Mean colony formation efficiency (%) was calculated from triplicate wells for each well in part B. Error bars are SDM of three independent experiments; **p<0.01 obtained by Student's t-test.
